# Complete Genome Sequence of *Ralstonia solanacearum* FJAT-1458, a Potential Biocontrol Agent for Tomato Wilt

**DOI:** 10.1128/genomeA.00070-17

**Published:** 2017-04-06

**Authors:** Deju Chen, Bo Liu, Yujing Zhu, Jieping Wang, Zheng Chen, Jiamei Che, Xuefang Zheng, Xiaoqiang Chen

**Affiliations:** Agricultural Bio-Resources Research Institute, Fujian Academy of Agricultural Sciences, Fuzhou, Fujian, People’s Republic of China

## Abstract

An avirulent strain of *Ralstonia solanacearum* FJAT-1458 was isolated from a living tomato. Here, we report the complete *R. solanacearum* FJAT-1458 genome sequence of 6,059,899 bp and 5,241 genes. This bacterial strain is a potential candidate as a biocontrol agent in the form of a plant vaccine for bacterial wilt.

## GENOME ANNOUNCEMENT

*Ralstonia solanacearum* is a soilborne plant-pathogenic betaproteobacterium with a wide host range and a wide geographic distribution. *R. solanacearum* infects more than 50 botanical families, including about 200 species, including tomato and potato, causing plant bacterial wilt (1–3), and is regarded as the second of the top 10 bacterial plant pathogens based on scientific/economic importance (4). *R. solanacearum* FJAT-1458 was isolated from living tomato vessel showing no toxicity to *Solanaceae* plants. *R. solanacearum* FJAT-1458 had penetrated the tomato tissue and stimulated resistance to protect the tomato plant from the virulence of *R. solanacearum*, indicating that this strain is a potential biological agent for the control of bacterial wilt in agricultural plants. More than 60 genomes of *R. solanacearum* which are plant pathogens have been deposited in GenBank (NCBI data). To further understand and advance the application of *R. solanacearum* FJAT-1458 for controlling plant bacterial wilt, we present its complete genome sequence, as well as its annotation.

The genome of *R. solanacearum* FJAT-1458 was sequenced using the PacBio RS II platform. A 10-kb single-molecule real-time (SMRT) bell library was prepared from sheared genomic DNA using a 10-kb template library preparation workflow. The DNA damage repair, end repair, and SMRT bell ligation steps were performed as described in the template preparation protocol with the SMRTbell template prep kit version 1.0 reagents (Pacific Biosciences, Menlo Park, CA, USA). SMRT sequencing was conducted on a PacBio RS II sequencing platform using C4 sequencing chemistry and P6 polymerase with one SMRT cell. Sequencing reads were *de novo* assembled following the Hierarchical Genome Assembly Process (HGAP) workflow (PacBioDevNet; Pacific Biosciences) as available in SMRT Analysis version 2.3.1 (5, 6).

The complete genome of *R. solanacearum* FJAT-1458 contains a 3,984,240-bp circular chromosome and a 2,075,659-bp megaplasmid, with G+C contents of 66.72% and 66.93%, respectively. Genome annotation was performed using the Prokaryotic Genome Automatic Annotation Pipeline (PGAP) (http://www.ncbi.nlm.nih.gov/books/NBK174280/). The chromosome contains 3,575 protein-coding genes and 54 tRNA, nine rRNA, and three other noncoding RNA (ncRNA) genes. The megaplasmid contains 1,490 protein-coding genes. We compared genome sequences of *R. solanacearum* FJAT-1458 with *R. solanacearum* GMI1000 by BLAST. Two copies of the insertion sequence (IS) element were screened in the FJAT-1458 genome, and one was inserted into a* phcA* gene. The *phcA* gene disruption caused no pathogenicity to host of* R. solanacearum* (7, 8). In conclusion, *R. solanacearum* FJAT-1458, a wild-type strain, is a potential candidate as a biocontrol agent or plant vaccine to combat bacterial wilt in tomato and other plants.

The availability of this genome will enhance the understanding of the role of avirulent *R. solanacearum* in inducing plant resistance to pathogens and facilitate its application for controlling bacterial wilt in agricultural plants.

### Accession number(s).

Genome information for the chromosome and megaplasmid of *R. solanacearum* FJAT-1458 has been deposited in GenBank under the accession numbers CP016554 and CP016555, respectively. The strain can be obtained from the microbiology laboratory of the Institute of Agricultural Biological Resource Research, Fujian Academy of Agricultural Science, Fuzhou, People’s Republic of China.
